# Development of a prognostic model related to mitochondria and programmed cell death-related genes in bladder cancer

**DOI:** 10.3389/fgene.2025.1615167

**Published:** 2025-12-04

**Authors:** Xuwei Zhao, Hongyao Liu, Chao Wang, Fuyu Guo, Bin Yang

**Affiliations:** 1 Shanxi Bethune Hospital, Shanxi Academy of Medical Sciences,Tongji Shanxi Hospital, Third Hospital of Shanxi Medical University, Taiyuan, China; 2 Tongji Hospital, Tongji Medical College, Huazhong University of Science and Technology, Wuhan, China

**Keywords:** bladder cancer, programmed cell death, prognosis, immune infiltration, mitochondrial

## Abstract

**Background:**

Various forms of programmed cell death (PCD) play a crucial role in regulating the development and spread of cancer, with mitochondria serving as key organelles involved in executing PCD. In this study, mitochondrial and PCD-related prognostic genes in bladder cancer (BLCA) were explored, and prognostic models were constructed.

**Methods:**

The GSE32894, GSE13507, and The Cancer Genome Atlas (TCGA)-BLCA datasets related to BLCA were retrieved. The intersection genes of differentially expressed genes (DEGs) between BLCA and control samples in TCGA-BLCA, mitochondrial-related genes (MRGs), and PCD-related genes (PCD-RGs) were obtained for univariate and multivariate Cox regression analysis. Prognostic genes were selected, and a prognostic model was developed. Then, the validity of the model was evaluated and subsequently validated in the GSE32894 dataset. To enhance the clinical precision of the model, a nomogram was developed and combined with clinical traits. Enrichment analysis, immune infiltration analysis, and drug sensitivity analysis were employed. Finally, the expressions of prognostic genes were verified in the GSE13507 and TCGA-BLCA datasets and by real-time quantitative polymerase chain reaction (RT-qPCR).

**Results:**

Based on the above analysis, five prognostic genes (*POLB*, *FASN*, *CASP9*, *VDAC2*, and *RHOT2*) were selected, and a prognostic model was constructed, which revealed a strong predictive capability for sample survival in both the TCGA-BLCA and GSE32894 datasets. Meanwhile, the risk scores of BLCA samples in TCGA-BLCA were calculated, and samples were divided into high- and low-risk categories based on the optimal threshold. Further analysis found that risk score, stage, and age were independent prognostic factors, and they were used to construct the nomogram. Thereafter, we observed that pathways (e.g., epithelial–mesenchymal transition (EMT) and inflammatory response) related to BLCA were markedly enriched in the high-risk patients, and the response to immunotherapy in high-risk patients was suboptimal. Importantly, the expression trends of FASN, VDAC2, and RHOT2 in the BLCA and control groups within the TCGA-BLCA and GSE13507 datasets, as well as in clinical samples, were consistent and significant.

**Conclusion:**

In this study, a novel prognostic model for bladder cancer was constructed based on POLB, FASN, CASP9, VDAC2, and RHOT2, which provided preliminary references for the prognostic evaluation of bladder cancer and subsequent studies related to its diagnosis and treatment.

## 1 Background

It is estimated that bladder cancer (BLCA) is the most common malignant tumor of the urinary system, presenting with symptoms such as hematuria (Malinaric et al., 2022). BLCA tends to be somewhat less common in women, although it can still occur as stealthily as in men. The tumors start as a few rogue cells in the bladder that have lost their way due to DNA mutations, and soon, these cells multiply uncontrollably (Tran et al., 2021), especially non-muscle invasive bladder cancer (NMIBC). NMIBC is prone to recurrence after surgical resection. It has up to a 50%–70% chance of recurrence in the first year (Grossmann et al., 2022). Even with intravesical instillations of chemotherapy and immunotherapy to keep the invader at bay, 15% of our cases advance to muscle-invasive bladder cancers (MIBCs) (Lee and Ham, 2020). MIBC is a strong fortress-section of the disease burden with a grim prognosis that can have a tremendous influence on patient quality of life and survival rates (Lee and Ham, 2020).

We mainly rely on imaging, cystoscopy, and urine cytology to detect recurrence early, but these methods are often not sensitive enough to effectively identify recurrence. Although recently developed blood and urine biomarkers are more powerful tools, challenges remain. Due to the lack of specific therapeutic targets, some advanced-stage patients still cannot receive effective treatment in the early stages (Dobruch and Oszczudlowski, 2021). Therefore, there is an urgent need to identify effective prognostic genes and new therapeutic targets to improve treatment outcomes and prognosis management for BLCA patients.

Mitochondria not only control signal transduction but also play a fundamental role in cell differentiation and programmed cell death (PCD) pathways (Dobruch and Oszczudlowski, 2021). PCD is an indispensable process in cell homeostasis and, thus, normal tissue development, covering various forms such as apoptosis, autophagy, ferroptosis, and pyroptosis. As a fundamental protection mechanism of the body against cancer, PCD is an important target for anticancer therapy that affects the process of tumor initiation, promotion, and metastasis. Mitochondria, the decisive executioners of PCD, harbor numerous pro- and anti-death factors (Peng et al., 2022; N et al., 2023; D'Arcy, 2019; Obeng, 2021). Their utility in oncology has been increasingly appreciated, with a dual function of tumor suppression and promotion. However, knowledge of how mitochondrial and PCD-related genes might relate to BLCA prognosis is lacking (Zhang et al., 2023a).

This study is distinct from previous research frameworks that have mostly focused on single pathways or a small number of genes. It is the first study to systematically integrate mitochondrial function with various forms of PCD, including apoptosis and autophagy. By employing multidimensional bioinformatics methods such as differential expression analysis and survival correlation analysis, the study aims to identify prognostic genes related to mitochondrial function and PCD and to construct relevant predictive models. These efforts provide new theoretical bases and scientific support for the diagnosis, targeted treatment, and clinical management of bladder cancer.

## 2 Methods

### 2.1 Data acquisition

The Gene Expression Omnibus (GEO) database (https://www.ncbi.nlm.nih.gov/geo/) was used to mine the transcriptomic sequencing data in the GSE32894 (GPL6947) and GSE13507 (GPL6102) datasets related to BLCA (Sjodahl et al., 2012; Lee et al., 2010). The dataset GSE32894 consisted of a total of 308 BLCA tissue samples, of which 224 samples with survival information were specifically chosen for the validation of the prognostic model. A total of 67 control samples, including 58 paracancerous controls and 9 normal controls, along with 165 primary BLCA samples from GSE13507, were selected to validate prognostic gene expression. The gene expression matrix and clinical traits of The Cancer Genome Atlas (TCGA)-BLCA were retrieved from the University of California, Santa Cruz (UCSC) Xena (https://xena.ucsc.edu/). A total of 430 samples were acquired from TCGA-BLCA, of which 405 cancer tissue samples (400 cases with available survival information) and 19 adjacent tissue samples were selected as the training set. The MitoCarta3.0 database (http://www.broadinstitute.org/mitocarta) offered the acquisition of 1,136 mitochondrial-related genes (MRGs). We also identified a total of 18 distinct patterns of PCD and key regulatory genes from literature, encompassing 1,964 genes and including apoptosis, autophagy, and alkaliptosis. After eliminating duplicates, we ultimately acquired 1,548 PCD-related genes (PCD-RGs) (Qin et al., 2023).

### 2.2 Differential analysis

To identify the differences in gene expression between BLCA tissues and control tissues, thereby laying a foundation for subsequent screening of prognostic genes, the differentially expressed genes (DEGs) between BLCA and control samples in the TCGA-BLCA dataset were identified by the DESeq2 package (version 1.38.3) (|log_2_ FC| ≥ 0.5, *p.*adj <0.05) (Love et al., 2014). The results were visualized through a volcano plot and heatmap, created using the ggplot2 package (version 3.3.6) and the heatmap3 package (version 1.1.9), respectively (Gustavsson et al., 2022; Zhao et al., 2014).

### 2.3 Functional analysis and protein–protein interaction network of intersection genes

The intersection genes of DEGs, MRGs, and PCD-RGs were obtained using the UpSetR package (version 1.4.0) and applied for subsequent analysis (Conway et al., 2017) to understand the biological functions and pathways related to intersection genes. The intersection genes were subjected to ID conversion using the clusterProfiler package (version 4.4.4). Then, functional annotations were made, focusing on the analysis of the Gene Ontology (GO) function and the Kyoto Encyclopedia of Genes and Genomes (KEGG) pathway (*p.*adj <0.05) (Wu et al., 2021). Visualization was enabled using the ggplot2 package (version 3.3.6). The STRING database (https://cn.string-db.org/) was utilized to explore the protein interactions among the intersecting genes, thereby constructing a protein–protein interaction (PPI) network (minimum required interaction score = 0.4).

### 2.4 Univariate and multivariate Cox regression analysis

The study aimed to identify prognostic genes significantly associated with the survival outcomes of patients with BLCA from the previously screened cross-genes and to construct a risk assessment model based on these genes. In TCGA-BLCA, the survival package (version 3.4-0) was utilized to conduct univariate Cox regression analysis on intersection genes, employing *p* < 0.05 and HR≠1 as criteria for identifying genes significantly associated with survival outcomes (https://CRAN.R-project.org/package=survival). Subsequently, a proportional hazards (PH) assumption test was performed on the results of univariate Cox analysis to confirm genes (*p* > 0.05). Stepwise regression method was employed to further identify prognostic genes, and multivariate Cox analysis was employed to construct risk models based on the prognostic genes using the formula: $risk\;score=\sum_{i=1}^{i}\left(coefi*expi\right)$ . The “coefi” represents the prognostic gene coefficients, and “expi” indicates the expression situation of prognostic genes.

### 2.5 Evaluation and validation of prognostic models

To evaluate the predictive performance of the BLCA prognostic model constructed based on the genes screened in the earlier stage and verify its generalizability, the BLCA samples from TCGA-BLCA were utilized for evaluating the prognostic model, while those from GSE32894 were employed for validating the prognostic model. The risk score of the samples was calculated using the formula, and based on its optimal threshold, the samples were divided into high- and low-risk categories. Then, the Kaplan–Meier (KM) curves for the two datasets were separately plotted by applying the survival package (version 3.4-0). The survival receiver operating characteristic (ROC) curves for 1–3 years were separately generated employing the timeROC package (version 0.4) in R (https://CRAN.R-project.org/package=survival, doi:10.1093). The area under the curve (AUC) >0.6 indicated that the model had a relatively good predictive performance for the survival status of the samples.

### 2.6 Independent prognostic analysis

The study aimed to clarify the association between risk scores and clinical characteristics of BLCA, identify independent factors influencing patient prognosis, and construct a tool for intuitively predicting patients’ survival outcomes. In TCGA-BLCA, the proportion of risk scores and the significance of the differences between groups for the five clinical traits (age, gender, pathologic_T, grade, and stage) were analyzed using the Wilcox test for two groups and the Kruskal test for multiple groups. The traits pathologic_M and pathologic_N were excluded from the analysis due to a higher number of missing values. Therefore, the significance of the risk score and five clinical traits on prognosis was analyzed by implementing univariate Cox regression, followed by performing a PH hypothesis test on significant traits. Subsequently, independent prognostic factors were obtained by multivariate Cox regression analysis. Finally, the nomogram for predicting the survival rate of patients from 1 to 3 years was created using the survival and rms packages (version 6.3-0) (Xu et al., 2023). Additionally, an evaluation of the nomogram was conducted using calibration curves and decision curve analysis (DCA).

### 2.7 Gene set enrichment analysis

Finally, gene set enrichment analysis (GSEA) was conducted using the clusterProfiler package (version 4.4.4) to explore differences in relevant biological functions and involved signaling pathways between two risk categories (Wu et al., 2021; Zhou et al., 2024). First, a differential analysis was conducted on high- and low-risk categories. The DEGs between the two risk categories were then sorted and enriched utilizing the GSEA function of the R language (*p*.adj <0.05). The Hallmark and KEGG gene sets were downloaded as background sets implementing the msigdbr package (version 7.5.1) (https://CRAN.R-project.org/package=msigdbr).

### 2.8 Immune analysis

In order to analyze the variation in immune infiltration between two risk categories, we implemented the ssGSEA algorithm provided by the GSVA package (version 1.44.5) to assess the enrichment scores of 28 immune cells in all samples within the TCGA-BLCA dataset, and then a Wilcoxon test was utilized to compare the variations in cell content between these two categories (*p* < 0.05) (Hanzelmann et al., 2013). Furthermore, Spearman correlation was applied to analyze the correlation between prognostic genes and risk scores with discrepant immune cell enrichment scores. In addition, the common immune checkpoint expressions of the two risk categories were compared, covering IDO1, PD-1, PD-L1, CD27, PD-L2, LAG3, TIM-3, ICOS, CTLA4, and LGALS9. The tumor immune dysfunction and exclusion (TIDE), dysfunction, and exclusion scores were computed for the samples in TCGA-BLCA, and their correlation with risk scores was computed to evaluate the immunotherapy efficacy of patients.

### 2.9 Drug sensitivity analysis and expression validation

The between-group differences in IC_50_ values of 198 drugs from Genomics of Drug Sensitivity in Cancer (GDSC, http://www.cancerrxgene.org/) were computed by the oncoPredict package (version 0.2), and the correlation between discrepant drugs and risk scores was assessed (*p* < 0.01) (Maeser DGruener RFHuang RS, 2021). The expressions of prognostic genes were definitively validated in both the TCGA-BLCA and GSE13507 datasets and in clinical samples. A total of 10 tissue samples were obtained from Shanxi Bethune Hospital, consisting of five control specimens and five BLCA specimens. Prior to sample collection, all participants provided signed informed consent forms, and the study received ethical approval from the Clinical Research Ethics Committee of Shanxi Bethune Hospital (SBQLL-2024-154).

The extraction and purification of total RNA were carried out using TRIzol (Ambion, Austin, United States), following the manufacturer’s instructions. Subsequently, cDNA was synthesized from the RNA using the SureScript First-Strand cDNA Synthesis Kit (ServiceBio, Wuhan, China). The resulting cDNA was then subjected to analysis using 2x Universal Blue SYBR Green qPCR Master Mix (ServiceBio) along with specific primer sequences (Additional file 1). Real-time quantitative polymerase chain reaction (RT-qPCR) amplification was performed under the following thermal cycling conditions: initial denaturation at 95 °C for 1 min, followed by 40 cycles of denaturation at 95 °C for 20 s, annealing at 55 °C for 20 s, and extension at 72 °C for 30 s. Data analysis employed the method known as 2^(-ΔΔCt), where GAPDH was used as an internal reference for normalization.

### 2.10 Statistical analysis

Data processing and analysis were carried out using R software (version 4.2.1), and difference analysis was conducted on the TCGA-BLCA dataset using the DESeq2 package (version 1.38.3). Enrichment analysis was performed using the clusterProfiler package (version 4.4.4), and Cox regression analysis was conducted using the survival package (version 3.4-0). ROC validation was conducted using the timeROC package (version 0.4), and nomograms related to independent prognostic factors were constructed using the survival and rms packages (version 6.3-0). GSVA analysis was performed using the GSVA package (version 1.44.5), expression analysis was conducted using the Wilcoxon test, and statistical significance was evaluated by setting a *p*-value threshold lower than 0.05.

## 3 Results

### 3.1 Screening of differentially expressed genes in BLCA and identification of mitochondria-PCD-related intersection genes

**FIGURE 1:**
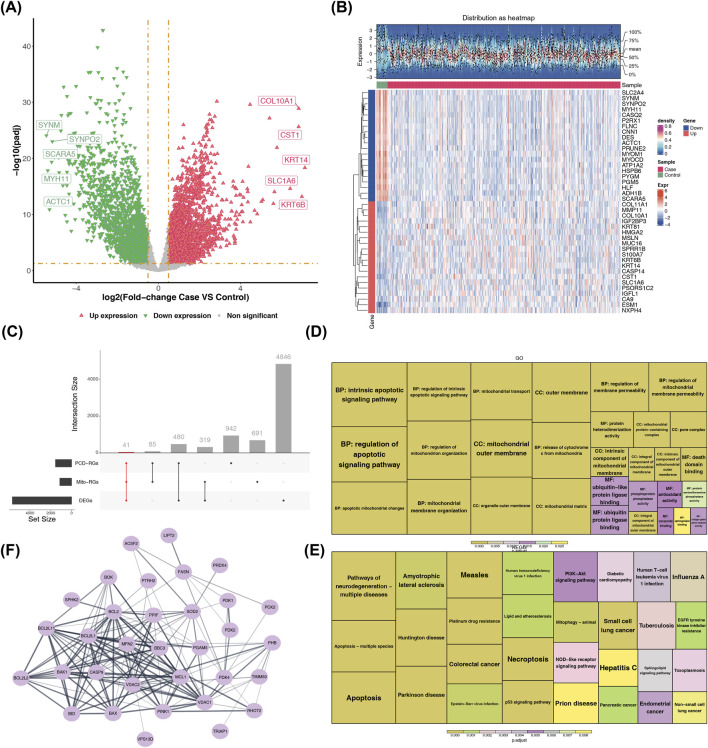
Differential expression, functional enrichment, and interaction network analysis in BLCA. **(A)** Volcano plot of DEGs between BLCA cases and controls. **(B)** Heatmap of DEGs. **(C)** UpSet plot showing overlaps between mitochondria and PCD-related genes and DEGs. **(D)** GO enrichment analysis of mitochondria and PCD-related DEGs. **(E)** KEGG pathway enrichment analysis of DEGs. **(F)** PPI network of key DEGs.

A total of 5,686 BLCA-DEGs were identified in the TCGA-BLCA dataset, with 3,266 upregulated and 2,420 downregulated genes (Figures 1A,B). The crossing of 5,686 BLCA-DEGs, 1,548 PCD-RGs, and 1,136 MRGs resulted in the identification of 41 intersection genes (Figure 1C). These intersection genes might be involved in both the regulation of programmed cell death and the remodeling of metabolic abnormalities in bladder cancer cells, providing potential candidate targets for subsequent exploration of tumor progression mechanisms and targeted intervention strategies.

### 3.2 Functional enrichment and PPI network analysis of mitochondrial-PCD-related intersection genes

In GO functions, these intersection genes were enriched in the intrinsic apoptotic signaling pathway, apoptotic mitochondrial changes, release of cytochrome c from mitochondria, and so on (Figure 1D). Additionally, they were associated with KEGG pathways, such as apoptosis, platinum drug resistance, and apoptosis—multiple species (Figure 1E). Notably, intersection genes were enriched in mitochondrial and PCD-related functions and pathways, which was consistent with our screening expectations. After excluding 8 genes without any interactions, we built a PPI network comprising 33 nodes and 147 pairs of interactions, such as VDAC2 with BAX and CASP9 with VDAC1 (Figure 1F). The results of these enrichment analyses, together with the construction of the PPI network, revealed the potential molecular network underlying the regulation of programmed cell death and metabolic abnormalities in bladder cancer, providing clues for in-depth analysis of BLCA progression.

### 3.3 Screening of mitochondria-PCD-related core prognostic genes, construction of prognostic model, and preliminary evaluation of efficacy

**FIGURE 2:**
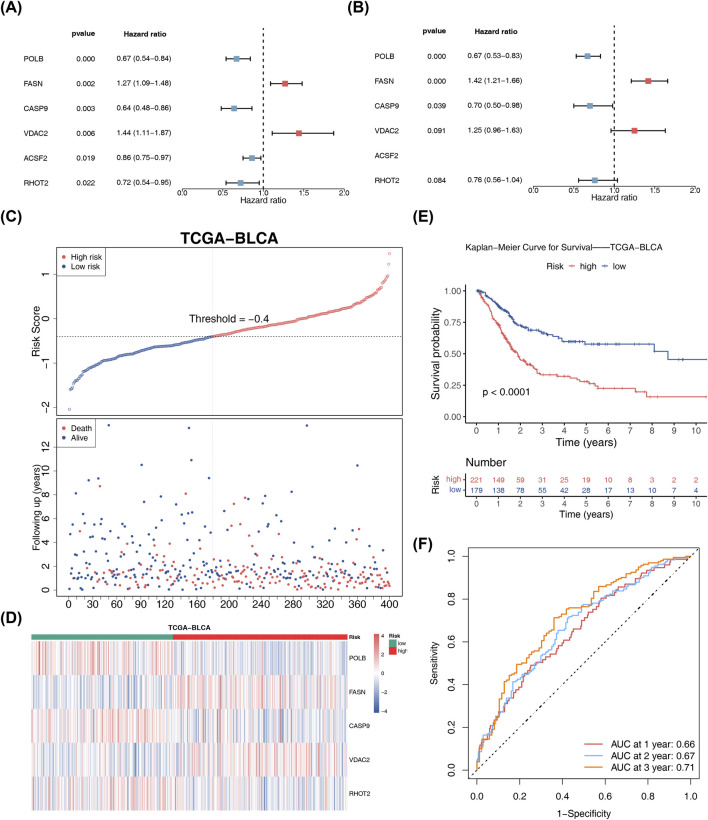
Prognostic gene analysis and risk score model validation in BLCA. **(A,B)** Results of univariate and multivariate Cox regression analyses, including the hazard ratios and significance levels for each gene. **(C)** Calculation of risk scores and the distribution of samples into high- and low-risk categories. **(D)** Expression patterns of prognostic genes: heatmap showing the differential expression of prognostic genes in the high-risk versus low-risk groups. **(E)** Kaplan–Meier survival analysis: Kaplan–Meier survival curves for high- and low-risk groups, with statistical significance indicated. **(F)** ROC curve analysis: ROC curves demonstrating the predictive accuracy of the risk score model for 1-year, 2-year, and 3-year survival.

After univariate Cox analysis and PH assumption testing of 41 intersection genes, a total of 6 prognostic-related genes were retained (Figure 2A). These six genes underwent further screening through stepwise regression to identify five prognostic genes (*POLB*, *FASN*, *CASP9*, *VDAC2*, and *RHOT2*), and a multivariate Cox analysis was then employed to construct a prognostic model (Figure 2B). The prognostic model was formulated as follows: riskScore = POLB × (−0.41) + FASN × 0.35 + CASP9 × (−0.36) + VDAC2 × 0.23 + RHOT2 × (−0.27). The risk scores of BLCA samples in TCGA-BLCA were calculated and divided into high- and low-risk categories according to their optimal threshold (−0.4). The high-risk category exhibited a higher number of deceased samples than the low-risk category, indicating that the risk score could provide a certain degree of prognostic evaluation for patients (Figure 2C). The expression of prognostic genes in categories is shown in Figure 2D. The KM curve revealed significant disparities in survival rates between the two risk categories, with the higher one exhibiting poor survival outcomes (Figure 2E). The model demonstrated a robust predictive ability for sample survival, as evidenced by the high AUC values of 0.66, 0.67, and 0.71 for the 1-, 2-, and 3-year survival in ROC curves, respectively (Figure 2F). The construction of this prognostic model provided a quantitative tool for the individualized risk assessment of bladder cancer patients. The five key genes involved might influence BLCA progression by regulating programmed cell death and metabolic reprogramming, offering potential molecular targets for the development of targeted therapeutic strategies.

### 3.4 Validation of the bladder cancer prognostic model in an external dataset (GES32894)

**FIGURE 3:**
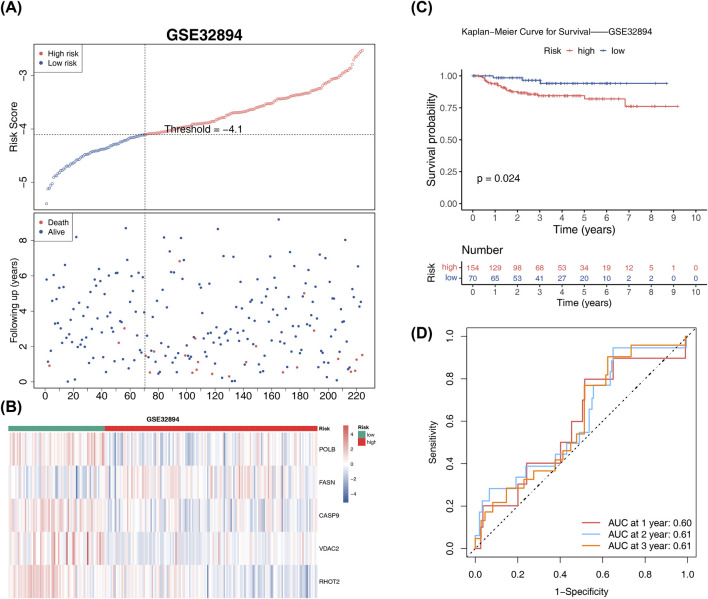
External validation of the prognostic model in the GSE32894 dataset. **(A)** Kaplan–Meier survival curves for high- and low-risk groups: Kaplan–Meier curves for the GSE32894 dataset, stratified by risk score. **(B)** Intergroup expression of prognostic genes in GSE32894. **(C)** Survival rate comparison: bar chart comparing survival rates between high- and low-risk groups at various time points, with *p*-values. **(D)** ROC curves for the prognostic model: ROC curves for the GSE32894 dataset, showing the model’s predictive accuracy for 1-, 2-, and 3-year survival.

The generalizability of the prognostic model was assessed by performing the same analysis in GES32894. The optimal threshold for all samples in this dataset was determined to be −4.1. The high-risk sample exhibited a higher mortality rate and significantly poorer survival, reaffirming the results observed in the TCGA-BLCA (Figures 3A–C). The AUC values of the 1- (0.60), 2- (061), and 3-year (0.61) survival in ROC curves exceeded 0.60, further confirming the reliability of the risk model (Figure 3D).

### 3.5 Association between prognostic risk score and clinicopathological features, and nomogram construction and validation

**FIGURE 4:**
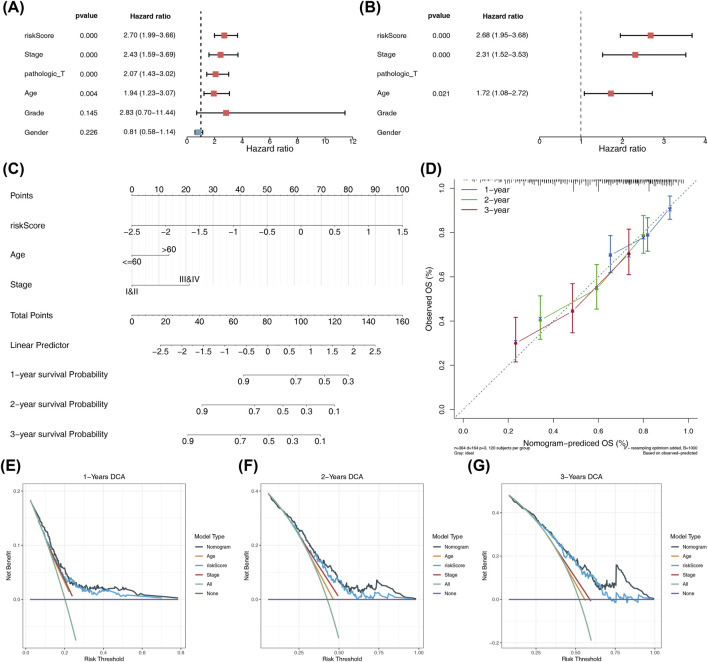
Assessment of independent prognostic factors and nomogram construction. **(A)** Cox regression analysis of clinical factors: forest map displaying the hazard ratios and significance of various clinical factors and risk scores. **(B)** Forest plot for multifactor Cox analysis. **(C)** Nomogram for survival prediction: heatmap-style nomogram integrating clinical factors and risk scores to predict 1-, 2-, and 3-year survival probabilities. **(D)** Calibration curves for the nomogram: line chart comparing predicted survival probabilities with actual observations for the nomogram’s predictive accuracy. **(E–G)** DCA for the nomogram: plot showing the decision curve analysis for the nomogram’s predicted survival probabilities over 1-, 2-, and 3-year periods.

The distribution of risk scores significantly differed among the age, grade, and stage groups, with risk scores increasing in correlation with age and disease severity in grade and stage (Additional file 2). In TCGA-BLCA, univariate Cox analysis, PH hypothesis test, and multivariate Cox analysis were implemented to determine the risk score, stage, and age as independent prognostic factors to construct the nomogram (Figures 4A–C). In the nomogram, as the total points increase, the patient’s survival probability decreases. The slope of the calibration curve approached 1, and the net proceeds of the model in the DCA were superior to those of the individual factors, indicating that the nomogram exhibited enhanced predictive efficacy (Figures 4D–G). By constructing a nomogram incorporating risk score, age, stage, and grade, the independent roles of these factors in the prognosis of bladder cancer patients were revealed. This provided a potential prognostic assessment tool for clinical practice and held certain clinical application value.

### 3.6 Hallmark and KEGG pathway enrichment analysis in BLCA patients in high- and low-risk groups

**FIGURE 5:**
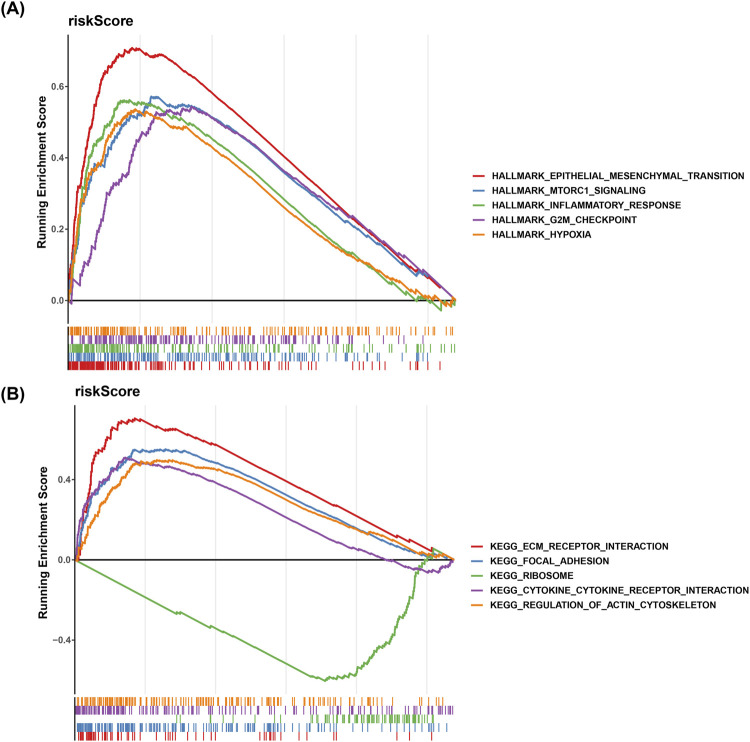
GSEA of high- and low-risk groups. **(A)** Hallmark pathway enrichment: line chart illustrating the enrichment of Hallmark pathways in high-risk versus low-risk groups. **(B)** KEGG pathway enrichment: line chart showing the enrichment of KEGG pathways in high-risk versus low-risk groups.

In the Hallmark background, epithelial–mesenchymal transition (EMT), MTORC1 signaling, and inflammatory response pathways were enriched in the high-risk patients (Figure 5A). KEGG pathways such as ECM–receptor interactions, cytokine–cytokine receptor interactions, and spot-of-adhesion were enriched in the high-risk category, while ribosome was enriched in the low-risk category (Figure 5B). The involvement of cytokine–cytokine receptor interactions, epithelial–mesenchymal transition, and inflammatory responses has been demonstrated in the pathogenesis of BLCA. This result revealed the potential roles of EMT, MTORC1 signaling pathway, and inflammatory response in the pathogenesis of BLCA, further expanding our understanding of the biological characteristics of BLCA.

### 3.7 Analysis of immune characteristics in BLCA patients in high- and low-risk groups

**FIGURE 6:**
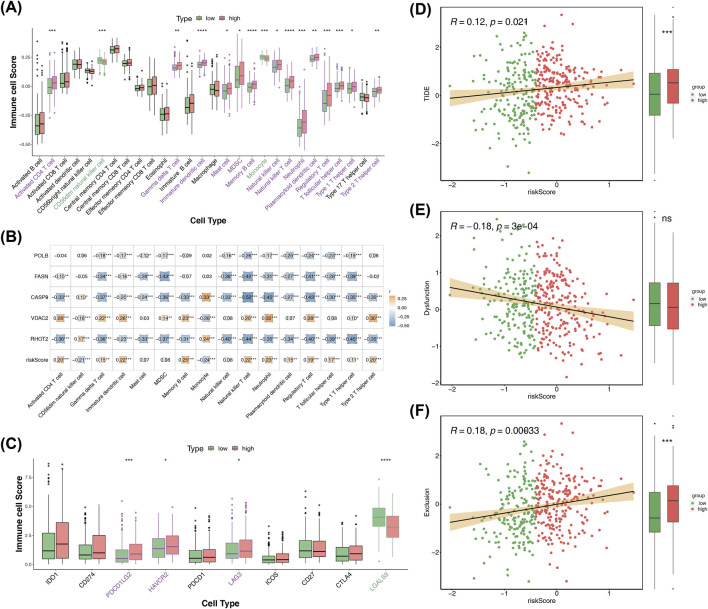
Immune landscape and correlation with risk scores. **(A)** Between-group differences in immune cell enrichment scores. **(B)** Prognostic genes, risk scores, and immune cell correlations. **(C)** Box plots of intergroup differences in immune checkpoints. **(D–F)** Correlation of TIDE-related scores with risk scores.

The two risk groups displayed remarkable differences in the levels of 16 immune cells, such as natural killer (NK) T cells, mast cells, γδT cells, activated CD4^+^ T cells, and CD56dim NK cells (Figure 6A). Interestingly, with the exception of CD56dim natural killer cells and monocytes, the levels of the remaining 14 immune cells were found to be higher in the high-risk patients, and the risk score was positively correlated with these cells (Figure 6B). This indicated that the prognosis of BLCA could be markedly influenced by the immune system. We also found that POLB, FASN, CASP9, and RHOT2 were negatively correlated with most of the differential immune cells, while VDAC2 was the opposite. Further analysis of immune checkpoint expression showed that PDCD1LG2, HAVCR2, LAG3, and LGALS9 were notably differentially expressed between the groups (Figure 6C). The analysis of the immunotherapy effect revealed that both TIDE and exclusion scores exhibited higher values in the high-risk patients, showing an increased trend following the increase of the risk score (Figures 6D–F). The findings implied a greater likelihood of immune evasion in patients at high risk. These results revealed the role of the immune system in the prognosis of bladder cancer and provided certain information for the subsequent optimization of immunotherapeutic strategies. The results may offer theoretical support for the formulation of individualized immunotherapy regimens for high-risk patients.

### 3.8 Drug sensitivity prediction in BLCA patients of high- and low-risk groups and expression validation of core prognostic genes

**FIGURE 7:**
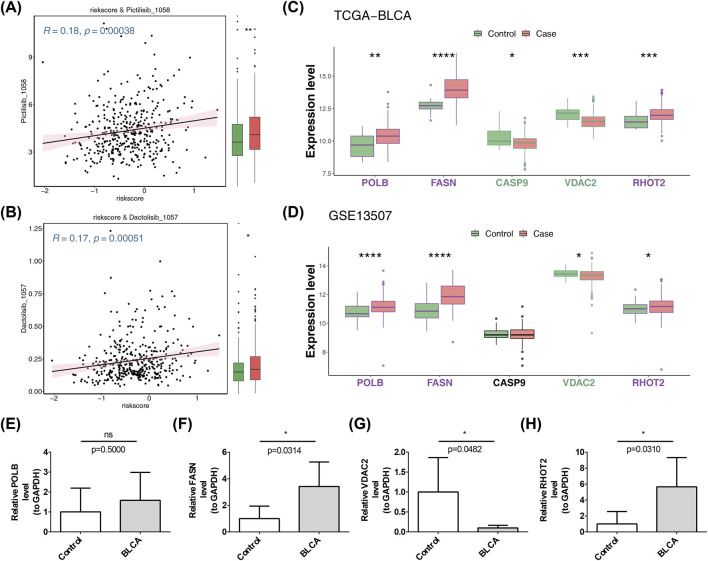
Drug sensitivity analysis and expression validation of prognostic genes. **(A)** Correlation with pictilisib sensitivity: scatterplot showing the correlation between risk scores and pictilisib sensitivity. **(B)** Correlation with dactolisib sensitivity: scatterplot illustrating the correlation between risk scores and dactolisib sensitivity. **(C)** Expression validation in TCGA-BLCA: bar chart comparing the expression levels of prognostic genes in control and BLCA cases from the TCGA-BLCA dataset. **(D)** Expression validation in GSE13507: bar chart comparing the expression levels of prognostic genes in control and BLCA cases from the GSE13507 dataset. **(E)** Expression validation of POLB in GSE13507: bar chart showing the relative expression level of POLB in control and BLCA cases from the GSE13507 dataset. **(F)** Expression validation of FASN in GSE13507: bar chart displaying the relative expression level of FASN in control and BLCA cases from the GSE13507 dataset. **(G)** Expression validation of VDAC2 and RHOT2 in GSE13507: bar chart presenting the relative expression levels of VDAC2 and RHOT2 in control and BLCA cases from the GSE13507 dataset. **(H)** Expression validation of RHOT2 in GSE13507: bar chart indicating the relative expression level of RHOT2, suggesting its role in BLCA.

A total of 29 drugs (including pictilisib, dactolisib, and erlotinib) exhibited prominent differences between the two risk categories, with a correlation *p*-value less than 0.01 for the association between drug IC_50_ value and risk score (Additional file 3). Among them, Figures 7A,B illustrate the results of pictilisib and dactolisib. Their low IC_50_ values indicated their potent inhibitory effect on tumor cells even at small drug concentrations. Except for CASP9 and POLB, the expression trends of the other three prognostic genes (FASN, VDAC2, and RHOT2) in the BLCA and control groups of the TCGA-BLCA and GSE13507 datasets were consistent, and the intergroup differences were noteworthy (Figures 7C–H). These results revealed the potential application value of specific drugs in the treatment of bladder cancer and provided a reference basis for individualized medication strategies based on risk scores.

## 4 Discussion

BLCA is pathologically divided into two major subtypes: NMIBC and MIBC (Tran et al., 2021; Grossmann et al., 2022; Lee and Ham, 2020). Although NMIBC can typically be treated through surgical resection, the recurrence rate remains extremely high, with 50%–70% of cases recurring within 1 year post-treatment. Despite the development of adjuvant strategies such as intravesical chemotherapy and immunotherapy, nearly one-sixth of NMIBC patients eventually progress to MIBC (Li et al., 2022; Gakis, 2020). MIBC is an aggressive disease that penetrates the muscular layer of the bladder wall, posing significant risks to both survival and quality of life.

In recent years, the critical roles of mitochondria and cell death pathways in cancer development have become increasingly recognized. However, few studies focus on the prognostic significance of mitochondrial and PCD-related genes in BLCA. To date, no comprehensive study has explored the prognostic value of mitochondria and 18 PCD-related genes in BLCA. This study aimed to fill that gap by systematically investigating the prognostic roles of these genes in BLCA, thereby providing novel insights into diagnostic and therapeutic strategies for BLCA.

Our research identified five key prognostic genes associated with BLCA—*POLB*, *FASN*, *CASP9*, *VDAC2*, and *RHOT2*—through comprehensive analysis of the GEO database. These genes are closely linked to mitochondria and PCD, suggesting that they may play significant roles in the prediction and treatment of BLCA. These findings provide valuable insights for future diagnostic and therapeutic strategies.

DNA polymerase β (POLB) is a crucial enzyme involved in the base excision repair pathway, which repairs single-strand breaks in DNA. Its proper function is vital for maintaining genomic stability, and dysregulation can lead to an accumulation of genetic mutations, which may contribute to carcinogenesis. Previous studies have highlighted the role of POLB in various malignancies. Existing studies have revealed the dual roles of POLB in tumors: on the one hand, research has confirmed that overexpression of POLB can directly promote tumor progression (Tan et al., 2016), which means that its high expression may be associated with increased tumor malignancy and poor survival prognosis. This is consistent with the results of our study, which show a high expression of POLB in patients with the disease. On the other hand, high expression of POLB can also reduce the accumulation of DNA damage in cells by enhancing the activity of the base excision repair pathway (Chen et al., 2024; Raper et al., 2021). The antitumor mechanisms of commonly used clinical chemotherapeutic drugs such as cisplatin and gemcitabine are precisely to inhibit proliferation by inducing DNA damage in tumor cells (Yu et al., 2017; Parvathaneni and Sharma, 2019). If the DNA repair function mediated by POLB is overly active, it may reduce the retention of damage induced by chemotherapeutic drugs, making cancer cells more resistant to subsequent treatment and thus increasing the risk of chemoresistance (Sakthivel and Hariharan, 2017). Therefore, it can be speculated that POLB may not only affect patient survival prognosis by directly promoting tumor progression but also participate in tumor treatment response by regulating DNA repair efficiency. Its overly active repair function may be a potential factor in weakening the effectiveness of chemotherapy and leading to resistance, ultimately indirectly affecting treatment prognosis. Thus, in-depth exploration of the specific mechanisms of action of POLB in bladder cancer is of great significance for optimizing bladder cancer treatment strategies and identifying new targets to overcome resistance. It also provides ideas for our future research directions.

FASN, as a key enzyme in fatty acid synthesis, is shown in this study to be highly expressed in tumors. It is known that tumor progression requires substantial energy and structural lipids (Li et al., 2017), and FASN can promote lipid accumulation (Deng et al., 2025). Lipid accumulation can affect the energy homeostasis and biomembrane synthesis of tumor cells, thereby promoting tumor progression (Chen et al., 2022). In various cancers, FASN is associated with high tumor grade and poor prognosis, and it has been suggested to contribute to the growth and invasion of bladder cancer (Dong et al., 2023). Therefore, it is speculated that its high expression may provide a material and energy basis for the malignant phenotype of bladder cancer by regulating lipid metabolism.

CASP9 acts as a “switch” in the cell death pathway, and its activation triggers a cascade of reactions leading to apoptosis (Baechler et al., 2019). Under normal circumstances, the release of cytochrome c from mitochondria leads to the formation of the apoptosome by binding with Apaf-1 and CASP9, which then activates downstream CASP3/7 to induce apoptosis (Kuwahara et al., 2003). This study indicates that its expression is downregulated, which may weaken the activation efficiency of the apoptotic pathway, thereby creating conditions for tumor cell survival.

VDAC2’s open channel can be likened to the “gatekeeper” of the mitochondrial “entrance and exit,” participating in the mediation of apoptosis (Yuan et al., 2021) and maintaining the stability of the mitochondrial membrane potential (Maldonado et al., 2013). ROS generation is affected by the stability of the mitochondrial membrane potential (Zhdanov et al., 2017). This study found that its low expression may affect the stable state of the mitochondrial membrane potential, thereby altering the level of ROS production and ultimately indirectly supporting tumor survival through ROS-mediated pathways.

The core function of RHOT2, as a mitochondrial Rho GTPase, is to regulate mitochondrial dynamics (fusion and fission) and transport (Nakornpakdee et al., 2025), and it can regulate mitochondrial dynamics by interfering with related pathways (Agarwal et al., 2019). It is known that mitochondrial dynamics have regulatory effects on tumors (Li et al., 2025). Although research on the function of RHOT2 in cancer is extremely limited at present, this study found that it can serve as a novel potential biomarker for bladder cancer, especially in relation to bladder cancer progression.

We used GSEA to identify altered pathways. Our analysis revealed significant enrichment in pathways related to cytokine–cytokine interactions, epithelial–mesenchymal transition, and inflammatory responses, which were most commonly observed in high-risk BLCA patients. These pathways serve as routes that cancer cells utilize to promote growth and metastasis while evading the immune system. The cytokine–cytokine receptor interaction pathway within the tumor microenvironment functions similarly to a communication system, facilitating interactions between cells. This pathway helps regulate the recruitment of immune cells to tissues, triggers inflammation, and influences tumor cell survival (Zhang et al., 2023b). Our study found that this pathway was significantly activated in high-risk patients, suggesting that inflammation plays a crucial role in tumor carcinogenesis and contributes to poor outcomes.

EMT has been implicated in the development of several types of cancer, including BLCA, promoting invasiveness and resistance to therapy (Xiao et al., 2022). Consistently, our results also indicated that the EMT pathway was enormously overlapped in high-risk patients. Significantly, GO analysis showed that the inflammatory response pathway was enriched in high-risk groups. This is important as chronic inflammation promotes the development of cancer. For example, inflammatory mediators have been implicated in BLCA-mediated survival and proliferation of the tumor cells (Yuan et al., 2019). In the future, the specific mechanisms of these pathways can be explored in depth. They can be considered potential therapeutic targets, which will have certain guiding significance for the clinical treatment and improved prognosis of bladder cancer.

Our study also investigated the behavior of tumors in relation to the immune response within these high-risk patients. High-risk patients in general have much more diverse immune cell populations than low-risk patients (Meng et al., 2022; Tan et al., 2023). Although the presence of active immune cells is crucial for an anticancer immune response, our study focused on developing a prognostic model that provides valuable insights into patient outcomes in BLCA. Our model demonstrates significant clinical value in prognostic prediction. High-risk patients show poorer outcomes than low-risk groups, with an AUC greater than 0.6, indicating good predictive performance. Univariate and multivariate Cox regression analyses confirmed that risk scores are independent predictors for BLCA patients. Additionally, we constructed a nomogram that incorporates risk scores along with clinical variables such as age and tumor stage. Calibration curves and DCA showed high predictive accuracy and clinical applicability for our model.

This study identified five prognostic genes related to mitochondria and PCD in BLCA that have the potential to serve as diagnostic and therapeutic targets. We also constructed a prognostic model for BLCA, providing new insights for guiding the diagnosis and treatment of patients with this disease. The study has some limitations. First, due to limited experimental resources and time, CASP9 has not been fully validated. As one of the core genes of the model, this omission may affect the comprehensiveness of the model and its subsequent potential for clinical promotion. Second, the model is mainly constructed based on mRNA expression data, while the execution of core biological functions, such as programmed cell death, depends on proteins. At present, there is a lack of systematic validation at the protein level, such as immunohistochemistry. The functional associations of the related genes must be further explored. In addition, the sample size for RT-qPCR validation is small, potentially affecting the robustness and generalizability of the results. As a potential clinical prognostic model, there is currently limited empirical support for experimental verification in aspects such as immune treatment response analysis. The study data mainly come from Western populations and may not fully represent the genetic and environmental diversity of other populations. The retrospective analysis design may introduce confounding factors, thereby affecting the accuracy of the association between gene expression and survival outcomes.

In follow-up studies, we will prioritize the validation of CASP9 and further experimental research, such as combining cell and animal models to carry out related protein expression validation and functional experiments, to clarify the expression and mechanisms of action of these prognostic gene proteins. At the same time, we will expand the clinical sample size and conduct extensive clinical studies with larger and more diverse multicenter patient cohorts. Through these measures, we can not only verify the reliability of the above genes as prognostic indicators but also further evaluate the prognostic efficacy and potential therapeutic value of the model, laying a solid foundation for the development of new diagnostic methods and therapeutic strategies for bladder cancer.

## 5 Conclusion

This study identified five prognostic biomarker genes related to mitochondrial function and programmed cell death in bladder cancer (*POLB*, *FASN*, *CASP9*, *VDAC2*, and *RHOT2*) and preliminarily constructed a prognostic model based on these genes. The model demonstrated some predictive potential for survival. Correlation analysis of risk scores and pathway enrichment analysis suggested that pathways such as cytokine–cytokine receptor interactions, epithelial–mesenchymal transition, and inflammatory response might be associated with bladder cancer progression. Immune infiltration analysis also provided preliminary insights into the characteristics of high-risk patients. Although meaningful correlations were obtained through multi-dataset bioinformatics analysis, the specific functional mechanisms of these biomarkers still need experimental validation. From a translational medicine perspective, further exploration of their potential for combined use with existing therapeutic approaches is needed.

In summary, this study provides preliminary data support for the exploration of prognostic biomarkers related to mitochondrial function and programmed cell death in bladder cancer. The relevant analysis results may offer some references for the mechanistic research and individualized treatment direction of bladder cancer. In the future, the prognostic value and potential therapeutic significance of these genes must be validated with larger and more diverse clinical cohorts.

## Data Availability

The datasets analysed during the current study are available in the Gene Expression Omnibus (GEO) database [GSE32894 (https://www.ncbi.nlm.nih.gov/geo/query/acc.cgi?acc=GSE32894) and GSE13507 (https://www.ncbi.nlm.nih.gov/geo/query/acc.cgi?acc=GSE13507)], the University of California Santa Cruz (UCSC) Xena (https://xena.ucsc.edu/), and MitoCarta3.0 database (http://www.broadinstitute.org/mitocarta). The original contributions presented in the study are included in the article. Further inquiries can be directed to the corresponding author.
