# Supplementary figures and images for: Development of a prognostic model related to mitochondria and programmed cell death-related genes in bladder cancer
Source: Front Genet. 2025 Dec 4;16:1615167. doi: 10.3389/fgene.2025.1615167 (PMC12713357; doi:10.3389/fgene.2025.1615167)

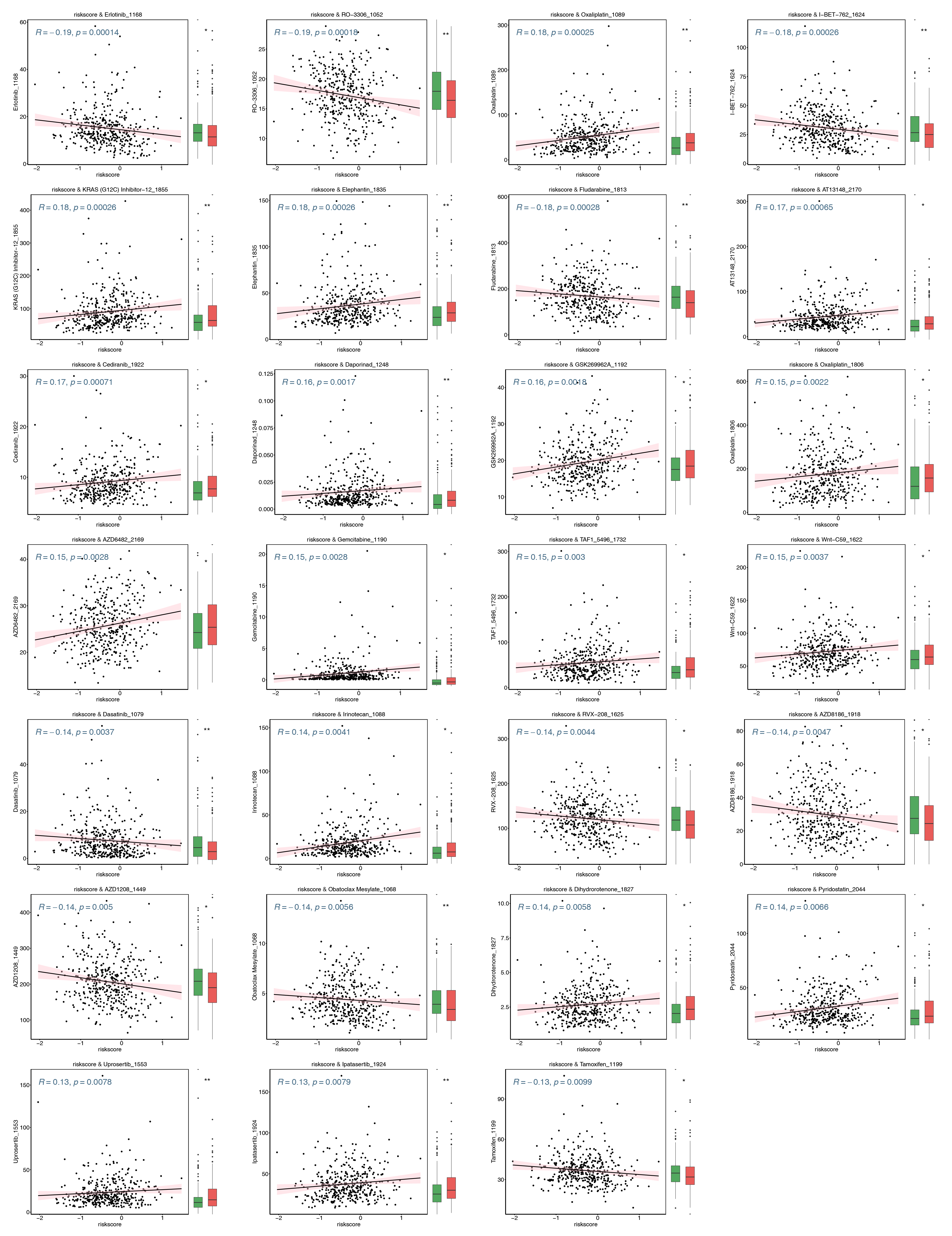

Supplement: Supplementary file 2 [file Image2.tif]

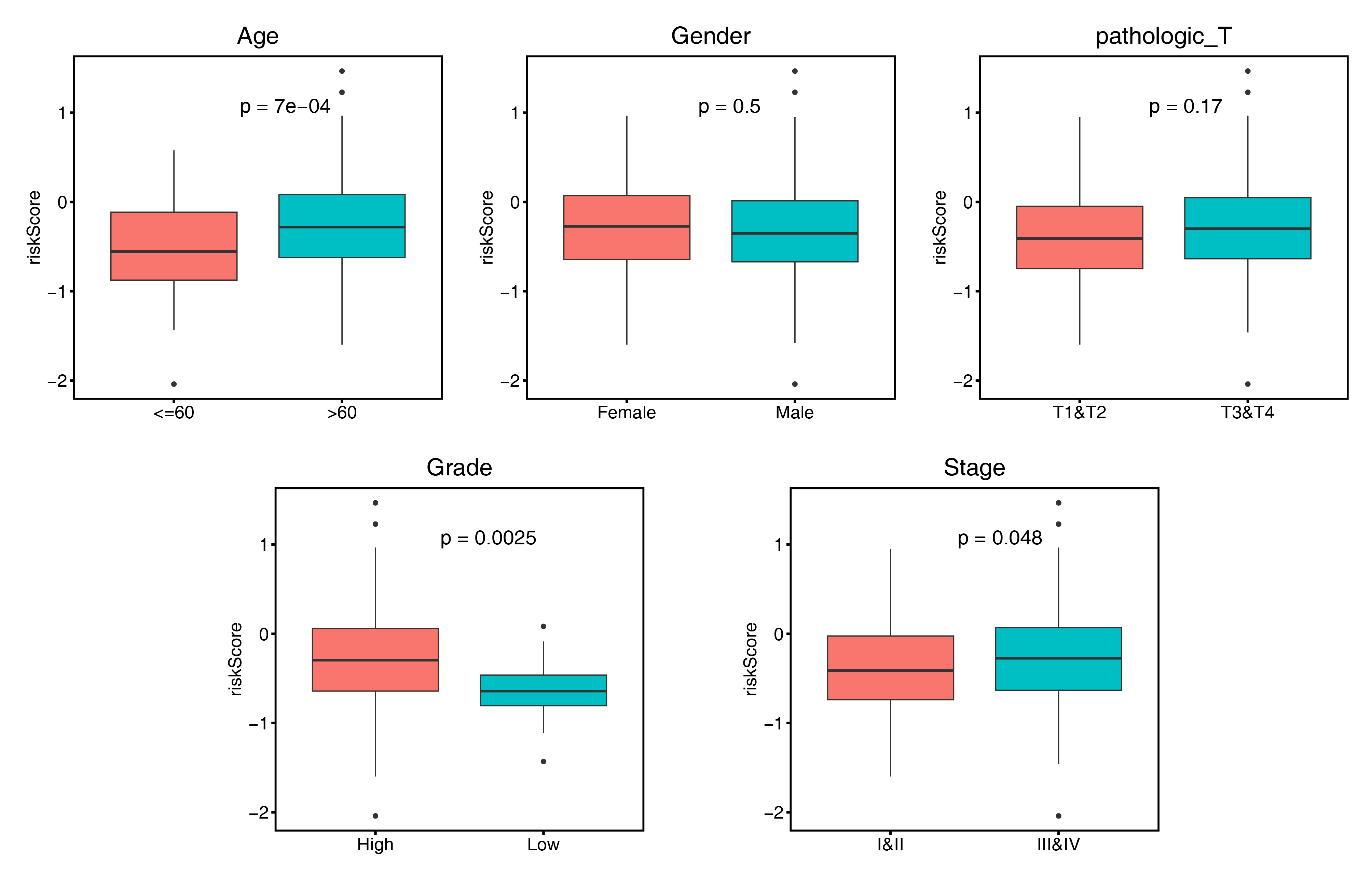

Supplement: Supplementary file 3 [file Image1.tif]
